# Pulmonary Hemosiderosis in a Child With Systemic Lupus Erythematosus: A Rare Presentation

**DOI:** 10.7759/cureus.7890

**Published:** 2020-04-29

**Authors:** Nikhil Rajvanshi, Swathi Chacham, Madhuradhar Chegondi, Jagdish P Goyal, Surjit Singh

**Affiliations:** 1 Pediatrics, All India Institute of Medical Sciences, Rishikesh, IND; 2 Pediatrics, University of Iowa Stead Family Children's Hospital, Iowa City, USA; 3 Pediatrics, All India Institute of Medical Sciences, Jodhpur, IND; 4 Pediatrics, Postgraduate Institute of Medical Education and Research, Chandigarh, IND

**Keywords:** child, pulmonary hemosiderosis, diffuse alveolar hemorrhage, systemic lupus erythematosus

## Abstract

Pulmonary hemorrhage is an uncommon manifestation in children and is often associated with systemic lupus erythematosus. We report a case of an adolescent girl who presented to our hospital with recurrent episodes of fever, cough, and breathlessness. Later on, she was diagnosed with pulmonary hemosiderosis as a manifestation of systemic lupus erythematosus. She was started on immunosuppressive therapy initially with prednisolone and subsequently with azathioprine and hydroxychloroquine, which improved the clinical status of the child.

## Introduction

Pulmonary hemosiderosis (PH) is a rare but serious manifestation of systemic lupus erythematosus (SLE). PH constitutes the triad of hemoptysis, iron deficiency anemia, and pulmonary infiltrates. Its pathogenesis is unknown, but it is considered to be immune-mediated. It is characterized by recurrent episodes of bleeding in alveoli followed by hemosiderin accumulation in alveolar macrophages and subsequent alveolar basement thickening and, finally, interstitial fibrosis [1]. It is a medical emergency and necessitates prompt diagnosis as a delay in diagnosis results in the worsening of pulmonary fibrosis.

## Case presentation

A 12-year-old girl child presented with complaints of fever, cough, and shortness of breath along with chest pain for the past 20 days. Her history was significant for similar symptoms requiring multiple hospitalizations that were treated as recurrent lower respiratory tract infections. The child received multiple blood transfusions for the last seven years for severe anemia. She had no history of joint pains, serositis, or symptoms suggestive of other organ system involvement. The child was admitted to the pediatric ward for further management.

Upon admission, the child was tachypneic, tachycardic, normotensive, and with oxygen saturation at 80% on ambient air. General examination revealed alopecia, malar rash, pallor, and grade III clubbing. Anthropometry was suggestive of chronic malnutrition, with weight and height for age 3 standard deviations below the mean. Systemic examination was positive for bilateral crackles, bronchial breathing, and grade II systolic murmur over the precordium. The child was kept on nasal cannula oxygen therapy and started on intravenous antibiotics after sepsis workup.

**Figure 1:**
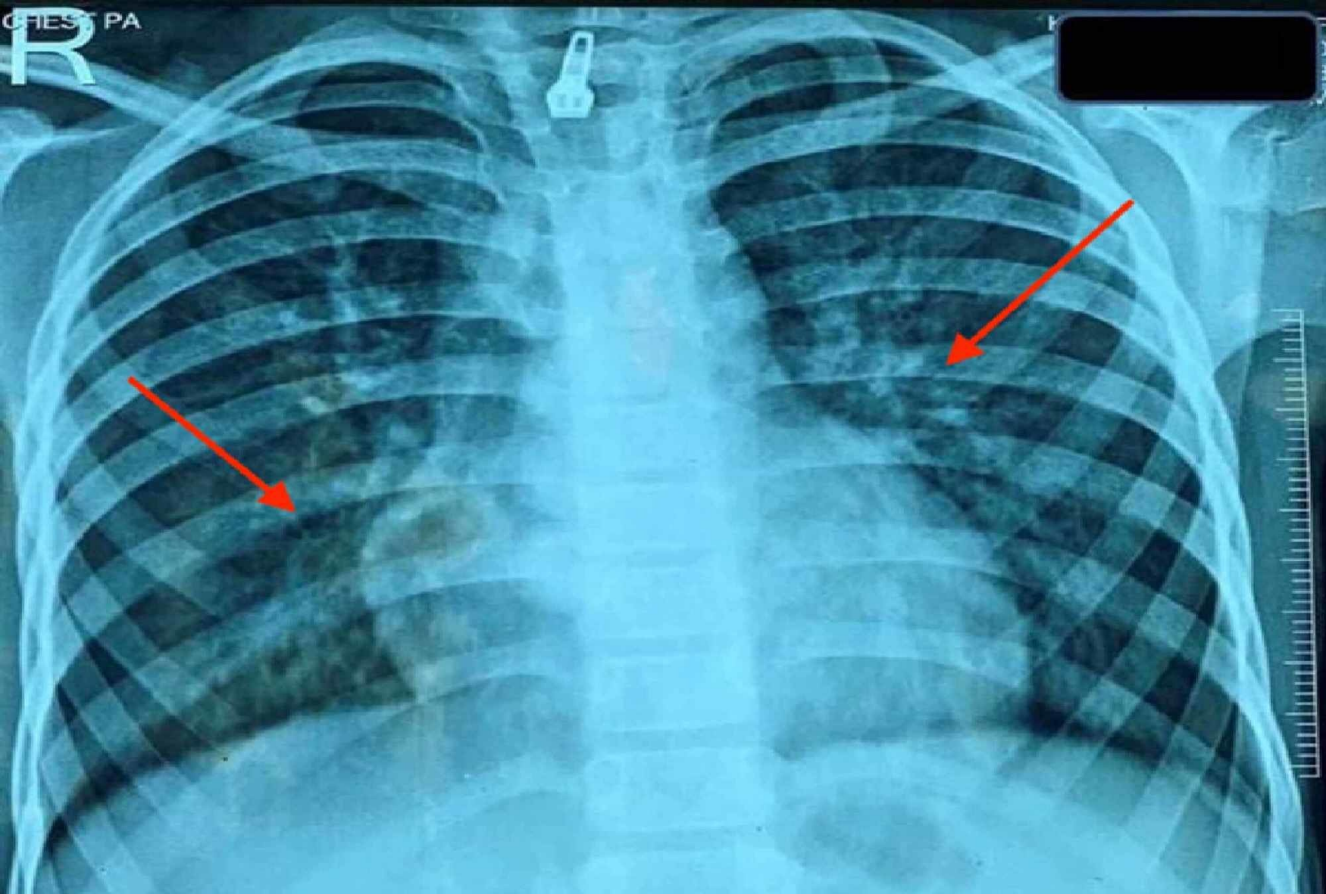
Chest X-ray showing diffuse infiltrates involving bilateral lung fields, predominantly perihilar regions (red arrows).

**Figure 2:**
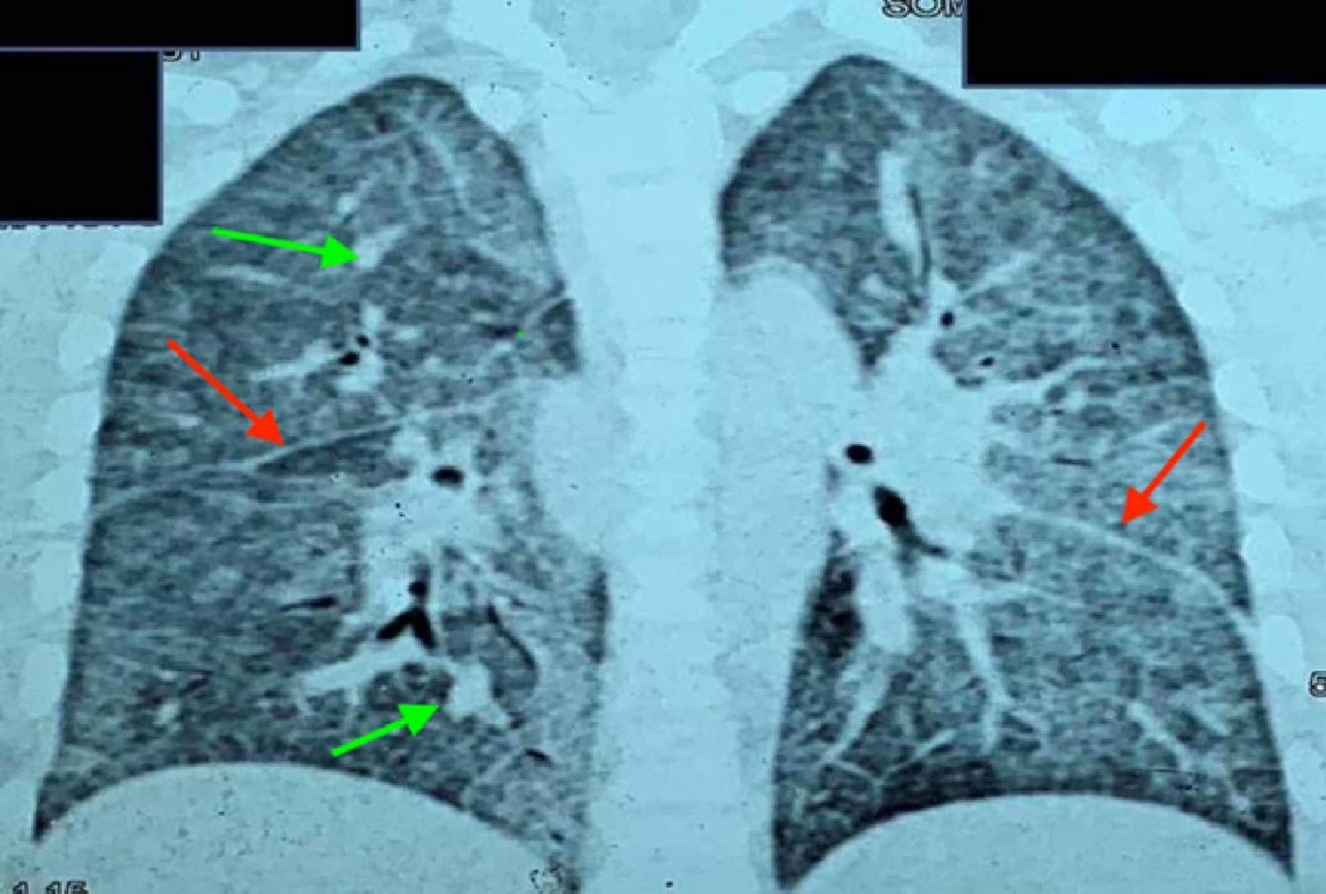
Coronal sections of HRCT of the thorax showing thickened interlobular septa (red arrows) with diffuse ground-glass opacities and nodular opacities (green arrows), suggestive of diffuse alveolar hemorrhage. HRCT, high-resolution computed tomography

Investigations revealed severe anemia with a hemoglobin level of 3 gm/dL and serum iron level of 11 mcg/dL (normal level: 50-120 mcg/dL). Workup for hemolytic anemia was negative. Given respiratory distress with grade III clubbing and diffuse infiltrates on chest X-ray (Figure 1), infectious etiology was considered, and workup including testing for tuberculosis was negative. High-resolution computed tomography (HRCT) was performed later, which showed diffuse alveolar hemorrhage (DAH; Figure 2),

 

Cardiac etiology and bone marrow involvement were ruled out with normal echocardiography and bone marrow examination. Further workup with bronchoalveolar lavage and gastric aspirate revealed iron-laden macrophages, which was consistent with PH. Urine analysis showed microscopic hematuria with mild proteinuria. With a possibility of the pulmonary-renal syndrome, workup was performed for SLE. Antinuclear antibody (ANA) was three-plus homogenous, and the remaining autoimmune workup was negative, including complement, anti-double-stranded DNA antibody (anti-dsDNA), anti-neutrophil cytoplasmic antibodies (ANCA), anti-glomerular basement membrane (anti-GBM) antibody, tissue transaminase (TTG) antibody, rheumatoid factor (RF), antiphospholipid antibody (APLA), and anti-beta-2-glycoprotein. Considering clinical features of alopecia and malar rash in the setting of ANA positivity, a probable diagnosis of SLE was made, and she was started initially on oral prednisolone and subsequently on azathioprine and hydroxychloroquine, which led to significant clinical improvement. The child also received 40 mL/kg of packed red blood cell transfusion during her hospital stay. She was discharged home in stable condition, and on follow-up visits, she continues to do well.

## Discussion

Our index case presented with PH with underlying probable SLE. Chronic pulmonary hemorrhage with PH is a rare but serious manifestation of SLE. The estimated prevalence of PH is less than 5% in children with SLE, with a mortality as high as 50% [2,3]. Childhood-onset SLE (cSLE) has a higher frequency of DAH as compared with adult-onset SLE and with a male predominance [3,4]. Other than PH, DAH also associates with various vasculitis, autoimmune, and platelet disorders [5]. Children with chronic pulmonary hemorrhage may only present with anemia requiring recurrent blood transfusions. Anemia is a universal finding in such cases [3]. In our case, the child presented with a history of recurrent lower respiratory tract infections requiring multiple blood transfusions. Dyspnea and fever are the most common clinical features followed by hemoptysis in children with PH [6,7]. Thrombocytopenia may also present with DAH [8]. In our index case, the child did not have hemoptysis or thrombocytopenia. Peripheral smear and DAT (direct antiglobulin test) can give a clue regarding SLE, but in our case, it was negative [3].

As PH presents with cough and respiratory distress, it is vital to rule out infectious etiologies. In our case, all the infectious workup including tuberculosis was negative. The most common finding on radiological imaging in children with DAH is a diffuse pattern of intra-alveolar bleed, as demonstrated by ground-glass opacities and septal thickening and as noted in our index case [9]. Once the diagnosis of PH has been made, a detailed workup needs to be performed to find out the underlying etiology to initiate appropriate treatment. The goal should be to evaluate for any extrapulmonary involvement even if the child is asymptomatic, as it will prompt further workup. The presence of microscopic hematuria with proteinuria in our patient made us suspicious of a multisystemic disease such as SLE or ANCA-related vasculitis. Anti-dsDNA is highly diagnostic for SLE in cases of renal involvement but it can also be negative, as highlighted by our case [3]. As shown in previous studies, anti-ds-DNA antibodies can be negative in up to 24.4% of SLE cases, and in up to 13.3% of cases, it can be positive during the initial course of illness with subsequent negativity [10]. Renal involvement has a positive correlation with anti-dsDNA positivity, whereas anti-dsDNA negativity has a higher incidence of serositis [10].

PH can be idiopathic or secondary to underlying autoimmune disorder [11]. A detailed workup needs to be performed before considering it to be idiopathic, as it will have a marked impact on the prognosis. The presence of ANCA or other autoantibodies is related to poor prognosis in children with PH [11]. Prognosis of these patients also depends on the duration of immunosuppression therapies as children on an extended course of immunosuppressive therapies have a better prognosis [12,13]. Other factors that have an impact on prognosis include the time of diagnosis and the presence of other co-morbidities such as anemia [13,14].

Literature on the management and long-term follow up of PH in cSLE patients is limited. Various immunomodulators have been used as a treatment modality. Corticosteroids are the mainstay of treatment and used as first-line agents. Steroid therapy has an impact on survival rates and also decreases the relapse rate of pulmonary bleeding episodes as well as also slows down the progression of pulmonary fibrosis in these cases [15]. In steroid-resistant cases, immunosuppression agents such as azathioprine and hydroxychloroquine are used. The use of these immunosuppressants has greatly improved the prognosis of children with PH, as shown by various studies [11,16-17]. Children with PH can achieve long-term remission on these agents, and monitoring of the clinical response can be done easily while on these agents [16]. We initially started prednisolone and subsequently added azathioprine and hydroxychloroquine, which improved the clinical status of the child. However, there is a need for further evidence to accept or reject a particular drug regimen, but early and aggressive treatment may have a better prognosis.

## Conclusions

Considering variable presentation and lack of overt symptoms such as hemoptysis, recognizing and treating PH is a challenging task. Even if the diagnosis of PH is made, a detailed evaluation is needed to find out the underlying etiology before labeling it as an idiopathic form of PH. SLE should be considered in PH with atypical presentation and with positive autoantibodies. Prompt diagnosis with early institution of aggressive therapy is key to a successful outcome.
